# Artificial intelligence in otolaryngology: current applications, limitations, and future perspectives

**DOI:** 10.1007/s00405-026-10560-x

**Published:** 2026-09-16

**Authors:** Eleni Litsou, Ioannis Tzotzis, Theodoros Malisovas, Asimakis Asimakopoulos, Georgios Psychogios

**Affiliations:** Department of Otorhinolaryngology, Head and Neck Surgery, University Hospital of Ioannina, University of Ioannina, Ioannina, Greece

**Keywords:** Artificial intelligence, Otolaryngology, Machine learning, Deep learning, Head and neck surgery, Rhinology, Otology, Laryngology

## Abstract

**Purpose:**

Artificial intelligence (AI) is increasingly integrated into modern otolaryngology practice and has emerged as one of the most rapidly evolving technologies in contemporary medicine. Recent advances in machine learning, deep learning, computer vision, and multimodal AI systems have accelerated the development of diagnostic and therapeutic applications across multiple otolaryngology subspecialties.

**Methods:**

This narrative review was conducted through a structured literature search of PubMed/MEDLINE, Scopus, and Web of Science databases. Relevant publications evaluating artificial intelligence applications in otolaryngology were identified using combinations of predefined keywords. Priority was given to peer-reviewed studies published in English that investigated clinically relevant diagnostic, prognostic, surgical, educational, or workflow-related applications of artificial intelligence within otolaryngology. Both retrospective and prospective studies, review articles, landmark methodological studies, and representative publications with substantial influence on the field were considered.

**Results:**

AI-assisted technologies have demonstrated promising clinical utility in diagnostic imaging, endoscopic assessment, audiology, rhinology, laryngology, vestibular medicine, surgical simulation, head and neck oncology, radiotherapy planning, and predictive analytics. Emerging evidence suggests that deep learning algorithms may enhance detection of sinonasal disease, facilitate automated image segmentation, predict lymph node metastasis, classify thyroid nodules, and support prognostic modeling in head and neck cancer patients. In addition, multimodal AI systems integrating radiologic, pathologic, molecular, and clinical data may further improve diagnostic precision and personalized treatment planning. Generative AI tools, including GPT-based large language models, have also demonstrated emerging applications in medical education, image interpretation, differential diagnosis, and clinical decision support.

**Conclusion:**

Nevertheless, important methodological and translational challenges continue to limit broader clinical implementation. Current challenges include limited external validation, retrospective study design, dataset heterogeneity, algorithmic bias, lack of transparency, patient privacy concerns, medico-legal uncertainty, and the potential risks associated with automation bias. This narrative review summarizes current clinical applications of artificial intelligence in otolaryngology, discusses the strengths and limitations of currently available technologies, and highlights future perspectives for responsible clinical integration. Current evidence suggests that AI may serve as a valuable adjunctive tool in otolaryngology practice; however, further prospective multicenter studies, standardized validation frameworks, and ethical oversight remain necessary before widespread adoption can be achieved.

## Highlights

- Artificial intelligence applications in otolaryngology are rapidly expanding.
- Deep learning models support emerging diagnostic and prognostic applications.
- Multimodal AI systems may support personalized patient management.
- GPT-based large language models demonstrate emerging utility in ENT practice.
- Ethical concerns and validation challenges remain major barriers to implementation.

## Introduction

Artificial intelligence (AI) has rapidly emerged as a transformative technology across multiple medical specialties, including otolaryngology [1–3]. Recent advances in machine learning (ML), deep neural networks, natural language processing, and large language models have accelerated the development of computational systems capable of supporting diagnostic interpretation, prognostic prediction, surgical training, and clinical decision-making [2–4]. Increasing availability of imaging datasets, electronic health records, endoscopic video archives, and integrated clinical information has further contributed to the expansion of AI-driven healthcare applications [4].

Within otolaryngology, integrated computational frameworks have been investigated across rhinology, otology, laryngology, vestibular medicine, sleep medicine, and head and neck oncology [1–3, 5]. Recent studies have described encouraging results in automated image interpretation, radiomic analysis, surgical skill assessment, pathology support, and personalized risk stratification [6–18]. Generative AI platforms and multimodal large language models have also shown emerging utility in clinical reasoning, image interpretation, and educational support [19–30].

Despite substantial enthusiasm surrounding these technologies, important barriers continue to limit broader clinical adoption. Concerns include algorithmic bias, limited reproducibility across institutions, ethical implications, patient privacy, dataset heterogeneity, and medico-legal responsibility [31–33].

The aim of this narrative review is to summarize current applications of AI in otolaryngology, discuss existing limitations, and highlight future perspectives for clinical integration.

Beyond algorithmic performance, increasing attention has recently focused on explainability, transparency, dataset fairness, and real-world integration of AI systems [31–34]. Explainable artificial intelligence (XAI) frameworks aim to facilitate clinician understanding of algorithmic decision-making processes and may enhance physician trust in AI-supported clinical workflows. In parallel, emerging reporting frameworks such as CONSORT-AI and SPIRIT-AI have emphasized the importance of standardized methodology, transparent validation, and prospective clinical evaluation for safe deployment of AI technologies in healthcare settings. These considerations are particularly important in otolaryngology, where substantial heterogeneity exists across imaging protocols, endoscopic techniques, acoustic datasets, and institutional treatment pathways [31–34].

Given the rapid evolution of both multimodal AI systems and generative models, otolaryngology represents a particularly dynamic field for future clinical AI integration.

## Literature search strategy

This narrative review was conducted through a structured literature search of PubMed/MEDLINE, Scopus, and Web of Science databases. Relevant publications evaluating artificial intelligence applications in otolaryngology were identified using combinations of the following keywords: “artificial intelligence”, “machine learning”, “deep learning”, “generative artificial intelligence”, “large language models”, “otolaryngology”, “head and neck surgery”, “rhinology”, “otology”, “audiology”, “laryngology”, “head and neck oncology”, “radiomics”, and “diagnostic imaging”.

Priority was given to peer-reviewed studies published in English that investigated clinically relevant diagnostic, prognostic, surgical, educational, or workflow-related applications of artificial intelligence within otolaryngology. Both retrospective and prospective studies were considered. Review articles, landmark methodological studies, and representative publications with substantial influence on the field were also included when appropriate.

Studies were selected based on their relevance to contemporary clinical applications, methodological significance, and contribution to the evolving evidence base of artificial intelligence in otolaryngology. Case reports, non-English publications, and studies with limited relevance to clinical otolaryngologic practice were generally excluded. Owing to the narrative nature of this review, formal systematic review methodology and quantitative meta-analysis were not performed.

### Hierarchy of evidence in otolaryngologic AI research

The current body of evidence regarding artificial intelligence in otolaryngology remains heterogeneous with respect to methodological quality and clinical maturity. For the purposes of interpretation, studies may be broadly categorized into three levels. First, exploratory or feasibility studies primarily evaluate proof-of-concept applications using retrospective datasets. Second, validation studies assess model performance through internal or external validation cohorts and provide stronger evidence regarding reproducibility and generalizability. Third, clinical implementation studies evaluate AI systems within real-world healthcare environments and assess workflow integration, clinical utility, and patient-centered outcomes. Although numerous exploratory investigations have been published, comparatively few AI applications have undergone rigorous external validation or routine clinical implementation [1, 3, 5, 15–18].

**Fig. 1:**
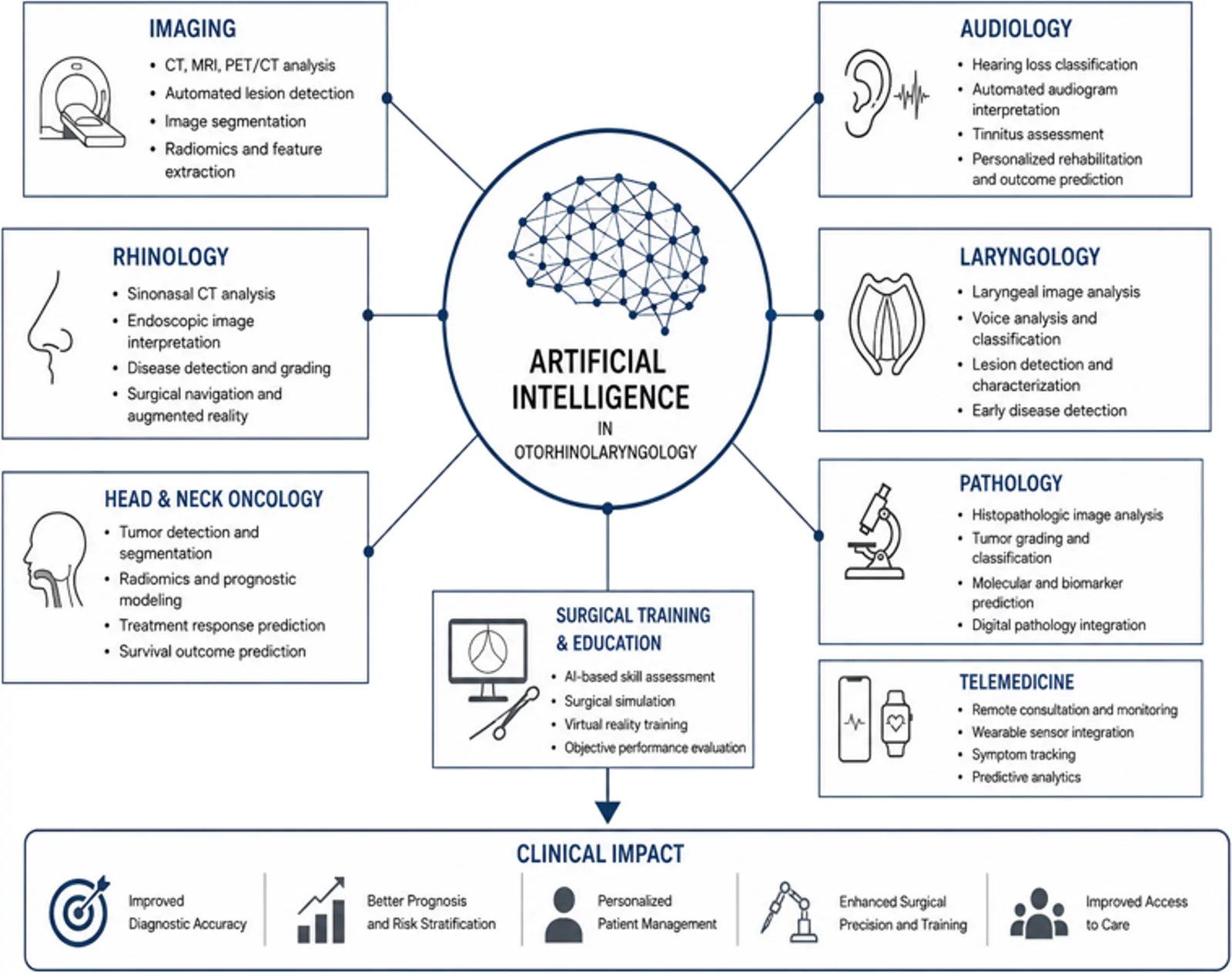
Overview of artificial intelligence applications across otolaryngology subspecialties, including rhinology, otology, laryngology, vestibular medicine, surgical education, and head and neck oncology

Representative clinical applications of AI across otolaryngology subspecialties are summarized in Table 1, while a schematic overview of current AI applications is presented in Fig. 1.

**Table 1:** Current applications of artificial intelligence in otolaryngology

Subspecialty	AI Application	Representative Technologies	Potential Clinical Benefit
Rhinology	Sinonasal CT analysis	Deep learning, convolutional neural networks	More accurate detection of sinus disease and automated image segmentation
Rhinology	Endoscopic surgical navigation	Computer vision, augmented reality	Greater intraoperative orientation and reduced complications
Otology	Hearing loss classification	Machine learning algorithms	More efficient audiologic interpretation and personalized rehabilitation
Otology	Middle ear disease diagnosis	GPT-4 Vision, multimodal AI	Automated image interpretation and diagnostic support
Vestibular Medicine	BPPV diagnostic modeling	Multimodal deep learning	Greater diagnostic accuracy and workflow optimization
Laryngology	Voice and laryngeal image analysis	Acoustic AI models, generative AI	Earlier disease detection and more accurate lesion classification
Head and Neck Oncology	Tumor detection and radiomics	PET/CT radiomics, transformer models	Prognostic stratification and personalized treatment planning
Thyroid Disease	Thyroid nodule classification	Multimodal AI, ultrasound deep learning	More reliable differentiation of benign and malignant lesions
Surgical Education	Technical skill assessment	Motion tracking, AI simulation	Objective assessment of surgical competency
Radiation Oncology	Automated target delineation	Large language models, deep learning	Lower interobserver variability and more efficient workflow integration

## Artificial intelligence in diagnostic imaging

**Fig. 2:**
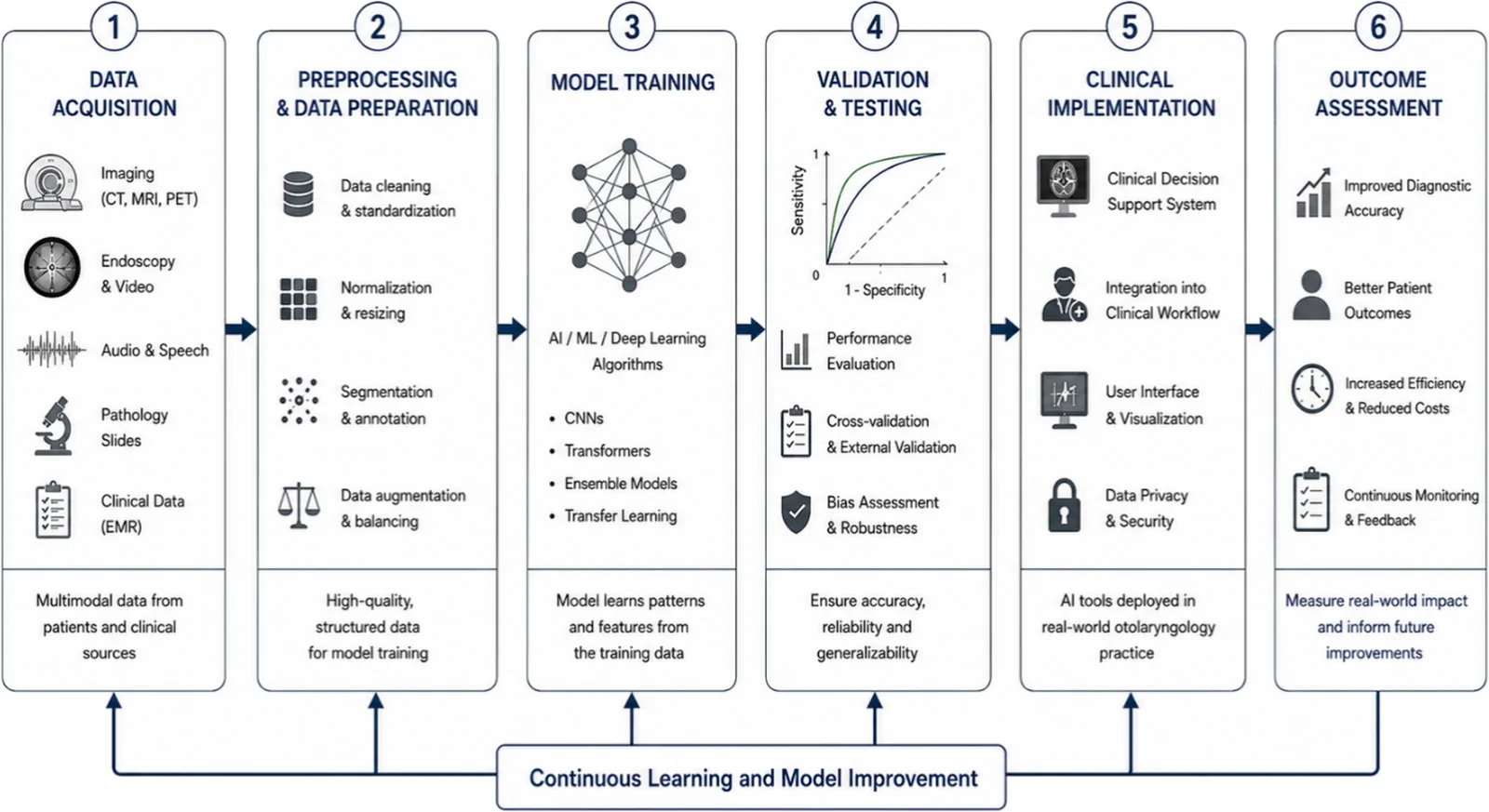
Simplified workflow of artificial intelligence integration in otolaryngology practice, including data acquisition, preprocessing, model training, validation, clinical implementation, and outcome assessment

AI is now being progressively incorporated into diagnostic imaging workflows within otolaryngology [3, 5–8, 35–37]. Advanced computational models have demonstrated promising potential in automated lesion detection, image segmentation, radiologic classification, and quantitative imaging analysis [3, 6–8, 35–37]. Convolutional neural networks and related deep neural architectures have demonstrated encouraging performance across multiple imaging modalities under experimental and validation settings, including computed tomography (CT), magnetic resonance imaging (MRI), endoscopy, and ultrasonography [3, 6–8, 35–37]. The general workflow of AI integration in otolaryngology practice is summarized in Fig. 2.

In rhinology, Jung et al. developed a convolutional neural network capable of automated segmentation of maxillary sinus lesions with high segmentation accuracy [6]. Kuwana et al. further reported favorable diagnostic performance using deep learning object detection technology for identifying maxillary sinus lesions on panoramic radiographs [7]. Automated quantitative sinus CT analysis has also shown encouraging clinical validity in studies evaluating chronic rhinosinusitis imaging [37].

Endoscopic image interpretation has also evolved rapidly in recent years. Li et al. developed and validated an endoscopic image-based neural network model for detection of nasopharyngeal malignancies, highlighting the emerging role of AI in endoscopic cancer screening [8].

Integrated computational frameworks combining imaging, clinical metadata, pathology, and temporal patient information may further enhance diagnostic performance compared with isolated single-modality systems. Soenksen et al. reported that healthcare AI models integrating text, imaging, tabular, and time-series data consistently outperformed single-source systems across multiple clinical prediction tasks, supporting the future incorporation of combined-data analytical architectures in otolaryngologic imaging applications [4].

In head and neck oncology, radiomics- and imaging-based predictive systems have actively been investigated for tumor detection, nodal staging, prognostic prediction, and treatment planning [9–18]. Advanced neural network approaches have shown encouraging capability in predicting occult lymph node metastasis in early tongue cancer and papillary thyroid carcinoma [9–11, 18]. PET/CT-based ML algorithms have also provided clinically relevant utility for HPV status classification and oncologic outcome prediction in oropharyngeal cancer [12, 13].

Although imaging-based AI applications have shown promising results, concerns regarding validation, reproducibility, and dataset heterogeneity persist. Many published imaging algorithms are trained using highly curated institutional datasets obtained under controlled conditions, potentially limiting real-world performance. Dataset shift, variability in imaging acquisition protocols, annotation inconsistency, and demographic imbalance may significantly reduce reproducibility across healthcare systems. In addition, algorithmic underdiagnosis bias has been reported in underserved patient populations, raising concerns regarding equitable deployment of AI-supported diagnostic systems in clinical practice [31, 32]. Current evidence remains primarily based on non-prospective investigations and requires larger multicenter validation studies before broader clinical incorporation can be recommended.

### Critical appraisal of current evidence

A critical limitation of the current literature is that the majority of published AI studies in otolaryngology are retrospective in design and rely on pre-existing datasets. While retrospective analyses are valuable for hypothesis generation and model development, they may overestimate real-world performance due to selection bias and controlled data environments. Prospective clinical validation studies remain relatively scarce across most otolaryngology subspecialties [1, 5, 15–18].

Another major concern is the limited availability of external validation. Many AI models demonstrate excellent performance within internal datasets but experience substantial declines in accuracy when applied to independent patient populations or institutions. This limitation raises concerns regarding model generalizability and clinical robustness [15–17].

Furthermore, overfitting remains an important methodological challenge. Complex deep learning models may learn dataset-specific patterns rather than clinically meaningful features, resulting in reduced performance when exposed to new data [15, 16]. Future investigations should prioritize prospective multicenter validation, transparent reporting standards, external validation cohorts, and standardized benchmarking methodologies to improve the reliability and clinical applicability of AI systems.

## Applications in audiology and otology

AI has shown considerable potential in hearing assessment, vestibular evaluation, and auditory rehabilitation. ML models can analyze audiometric patterns, classify hearing loss, and assist in cochlear implant optimization.

Law reviewed the application of AI to acoustic data in otolaryngology and highlighted the growing role of AI-assisted voice and hearing analysis systems [38]. Recent multimodal AI models have also been explored for benign paroxysmal positional vertigo diagnosis and middle ear disease classification [22, 24].

Generative AI systems with image recognition capabilities have provided promising feasibility in otologic image interpretation. Noda et al. investigated GPT-4 Vision for middle ear disease classification and reported encouraging diagnostic performance [24]. Similarly, multimodal AI systems integrating clinical and imaging data have shown potential in thyroid lesion classification and vestibular diagnostics [22, 23].

Collectively, current evidence suggests that AI applications in audiology and otology are particularly promising for pattern recognition, image interpretation, and decision support. However, most published studies remain exploratory or validation-based investigations, and broader prospective evaluation is required before routine clinical implementation can be recommended [22, 24, 38].

Wearable technologies and AI-assisted hearing rehabilitation systems may further enhance personalized patient care in audiology. However, variability in audiologic datasets and lack of standardized validation protocols continue to limit widespread implementation.

## Applications in rhinology and endoscopic surgery

AI technologies have also gained increasing attention in otolaryngology surgical training and simulation-based education [1, 39–45]. ML systems incorporating motion tracking, eye-tracking analysis, force measurements, and video-based assessment may contribute to more objective evaluation of technical skills compared with traditional subjective assessment methods [39–45].

Ahmidi et al. developed automated systems capable of differentiating surgical expertise levels during septoplasty and endoscopic sinus surgery using tool-motion analysis and eye-tracking data [44, 45]. Additional investigations have reported encouraging results using AI-supported simulation environments for standardized assessment of psychomotor skills and procedural competence [39–43].

Recent studies suggest that AI-based educational platforms may contribute to standardized trainee assessment, accelerated learning curves, and individualized performance feedback. Xiao et al. reported that AI systems achieved classification accuracies ranging from 66% to 100% across multiple simulation environments using motion, force, and video-based analyses [1]. Overall, AI-assisted surgical training systems appear most effective for objective performance assessment and automated feedback generation; however, evidence regarding their impact on real-world surgical competency and patient outcomes remains limited [1, 39–45].

Although AI-supported surgical education has attracted growing interest, important methodological and validation challenges persist. Many currently available studies are based on simulation datasets with relatively small participant cohorts, potentially limiting reproducibility in real operative settings. Furthermore, standardization of performance metrics, integration into surgical curricula, and prospective validation across institutions remain insufficiently established.

## Applications in head and neck oncology

AI applications in head and neck oncology have expanded substantially in recent years, particularly in tumor detection, radiomic analysis, prognostic modeling, treatment planning, and histopathologic interpretation [9–27]. Advanced AI systems integrating imaging, clinical, molecular, and pathologic data have shown promising performance in improving diagnostic accuracy, risk stratification, and personalized oncologic management.

Radiomics combined with ML has widely been investigated for prediction of lymph node metastasis, tumor behavior, treatment response, and survival outcomes in head and neck malignancies [9–18]. Qiang et al. developed a deep learning prognostic system for locoregionally advanced nasopharyngeal carcinoma that achieved strong predictive capability for survival outcomes [17]. Similarly, cross-institutional neural network models developed by Le et al. achieved encouraging prognostic performance in patients with head and neck cancer using self-attention architectures [16]. Kazmierski et al. further reported that multi-institutional deep learning and radiomic frameworks may facilitate outcome prediction and generalizability in head and neck oncology [15].

In oral and thyroid oncology, multiple imaging-based predictive systems have shown clinically relevant capability for identifying occult lymph node metastasis and prognostic stratification [9–11, 18]. Han et al. developed a CT-based integrated computational model for preoperative prediction of occult lymph node metastasis in early tongue cancer [9]. Wang et al. and Miao et al. similarly reported encouraging performance in predicting cervical lymph node metastasis in papillary thyroid carcinoma using deep learning and multimodal imaging systems [10, 11]. Yu et al. further reported that integrated multitask AI frameworks may predict molecular heterogeneity, nodal metastasis, and prognosis in papillary thyroid carcinoma [18].

Positron emission tomography/computed tomography (PET/CT)-based ML algorithms have also provided clinically relevant utility in oropharyngeal cancer. Woo et al. developed a predictive model using PET/CT-derived metabolic parameters for classification of human papillomavirus (HPV) status in oropharyngeal squamous cell carcinoma [12]. Ma et al. subsequently introduced a transformer-based PET/CT model capable of multi-outcome prediction in oropharyngeal cancer patients [13].

AI has additionally shown emerging applications in histopathology and endoscopic oncologic imaging. Mohseni et al. evaluated the utility of ChatGPT for differential diagnosis support in head and neck histopathology and reported encouraging diagnostic assistance capability [26]. Sievert et al. demonstrated the feasibility of GPT-4 Vision for identification of malignancy using confocal laser endomicroscopy in oropharyngeal lesions [21]. Liang et al. further evaluated multimodal large language models for image interpretation in laryngeal cancer surgery and reported variable but increasingly validated performance across different generative AI systems [30].

Radiotherapy planning and supportive oncologic care represent additional emerging fields of AI incorporation. Rajendran et al. developed large language model-augmented systems for automated delineation of treatment targets in head and neck radiotherapy, potentially facilitating workflow optimization and treatment standardization [46]. Dohopolski et al. also reported that deep learning models may predict the need for aggressive nutritional supplementation during head and neck radiotherapy [47].

Representative high-impact studies evaluating artificial intelligence applications in otolaryngology are summarized in Table 2.

**Table 2:** Representative high-impact studies on artificial intelligence applications in otolaryngology

Study	Year	Study Design	Sample Size	Subspecialty	AI Modality	Clinical Application	Performance Metric	Validation Type	Key Findings	Main Limitations
Surgical Training and Technical Assessment										
Ahmidi et al. [45]	2012	Experimental	378 ESS task trials (7 experts, 13 novices)	Rhinology / Surgical Training	Eye-tracking + ML	Objective surgical skill assessment	Accuracy: 94.6% (experts), 88.6% (novices)	IV	Accurate differentiation of surgeon skill levels.	Simulation-based; limited EV.
Ahmidi et al. [44]	2015	Observational	Intraoperative septoplasty procedures	Rhinology / Surgical Training	Tool-motion analysis	Automated intraoperative skill evaluation	Accuracy: 91%	IV	Objective assessment of surgical expertise during septoplasty.	Single-center; limited EV.
Kasa et al. [39]	2022	Experimental	72 surgical trainees and faculty	Surgical Training	Multimodal DL	Automated technical skill assessment	Expert-level performance (ICC, MSE)	IV	Expert-level performance in surgical skill assessment.	Simulation-based; limited clinical validation.
Khalid et al. [41]	2020	Experimental	103 surgical video clips from 8 surgeons	Surgical Training	DL/ video analysis	Surgical action recognition and performance evaluation	Precision: 0.97; Recall: 0.98	IV	Automated recognition of surgical actions and competence.	Small cohort; limited EV.
Mirchi et al. [42]	2020	Experimental	50 participants (28 skilled, 22 novice)	Surgical Training	Explainable AI + ML	Virtual operative assistant for simulation-based training	Accuracy: 92%; specificity: 82%; sensitivity: 100%	Experimental validation	Automated feedback and proficiency benchmarking during simulation.	Simulation-based; early-stage implementation.
Xiao et al. [1]	2025	Scoping review	34 included studies	Surgical Training	ML / DL/ computer vision	AI-assisted surgical skills evaluation	Accuracy 60–100% across motion-, force-, and video-based assessments	Literature synthesis	AI-supported objective skill assessment and trainee feedback.	Study heterogeneity; limited prospective validation.
Rhinology and Imaging Analysis										
Jung et al. [6]	2021	Retrospective	83 CBCT cases	Rhinology	Deep active learning / CNN	Maxillary sinus lesion segmentation	DSC: 0.75–0.77	IV	Accurate sinus lesion segmentation with reduced annotation burden.	Small dataset; no multicenter validation.
Kuwana et al. [7]	2020	Retrospective	1174 maxillary sinuses	Rhinology	DL object detection	Detection and classification of maxillary sinus lesions	Accuracy: 90–100%; Sensitivity: 100%	IV	Accurate maxillary sinus lesion detection and classification.	Single-modality dataset; no EV.
Massey et al. [37]	2022	Validation study	88 sinus CT scans	Rhinology	DL	Automated quantitative sinus CT analysis	Correlation with Lund-Mackay score: ρ = 0.85	Clinical IV	Objective CT analysis correlated with disease severity scores.	Retrospective; no prospective multicenter validation.
Amanian et al. [5]	2023	State-of-the-art review	59 included studies	Rhinology	Multiple AI techniques	Imaging analysis, disease classification, surgical navigation, and outcome prediction	Literature synthesis	Literature synthesis	Broad diagnostic, prognostic, and surgical applications in rhinology.	Study heterogeneity; limited prospective validation.
Otology, Laryngology, and Vestibular Medicine										
Law [38]	2024	Narrative review	Review of published acoustic AI studies	Otology / Laryngology	Deep neural networks	Acoustic signal analysis and voice/hearing assessment	Literature synthesis	Literature synthesis	AI-supported acoustic analysis for otologic and laryngologic applications.	Early-stage implementation; limited prospective validation.
Lu et al. [22]	2024	Diagnostic modeling study	518 patients with BPPV	Vestibular Medicine	Multimodal DL	Automated BPPV diagnosis	Accuracy: 81.7%	IV	Multimodal integration improved automated BPPV diagnosis.	Single-center; limited EV.
Noda et al. [24]	2024	Validation study	305 otoscopic images	Otology	GPT-4 Vision	Middle ear disease classification	Accuracy: 82.1%	Comparative validation	Multimodal GPT-4 Vision supported middle ear disease classification.	Proof-of-concept; limited EV.
Maniaci et al. [20]	2024	Prospective comparative study	40 patients	Laryngology	GPT-4	Laryngeal clinical image interpretation	ICC: 0.965	Comparative validation	GPT-4 supported laryngeal diagnostic assessment.	Small cohort; early-stage validation.
Head and Neck Oncology										
Li et al. [8]	2018	Retrospective with prospective comparison cohort	28,966 endoscopic images	Head & Neck Oncology	DL	Detection of nasopharyngeal malignancy	Accuracy: 88.7%	Prospective comparison validation	DL outperformed expert assessment in nasopharyngeal cancer detection.	Single-population cohort; limited EV.
Han et al. [9]	2024	Retrospective	125 patients with early-stage tongue cancer	Oral Oncology	Multiomics integrated AI model	Prediction of occult lymph node metastasis	AUC: 0.949; Sensitivity: 100%	IV	Integrated AI improved prediction of occult nodal metastasis.	Retrospective; single-center; no EV.
Wang et al. [10]	2024	Retrospective	611 patients with PTMC	Thyroid Oncology	Ultrasound-based DL	Prediction of cervical lymph node metastasis	AUC: 0.65	IV	Moderate performance in predicting lymph node metastasis.	No added benefit; no EV.
Miao et al. [11]	2025	Multicenter retrospective study	661 patients with PTC	Thyroid Oncology	Multimodal ultrasound-radiomics AI model	Prediction of central lymph node metastasis	AUC: 0.818 (EV)	Multicenter EV	Multimodal AI improved prediction of nodal metastasis.	Retrospective despite multicenter EV.
Woo et al. [12]	2023	Retrospective	126 OPSCC patients with 19 EV cases	Oropharyngeal Oncology	PET/CT-based ML	HPV status classification	AUC: 0.78 (EV)	EV	Multimodal ML improved HPV status classification.	Small EV cohort; retrospective.
Ma et al. [13]	2024	Retrospective	400 patients with OPSCC	Oropharyngeal Oncology	Transformer-based AI	Multi-outcome survival prediction (LRC, DMFS, OS)	OS C-index: 0.77	IV	Transformer-based AI improved prognostic stratification.	Retrospective; single-center; no EV.
Vollmer et al. [14]	2024	Retrospective	406 patients with OSCC	Oral Oncology	Multimodal AI-based pathogenomics	Multimodal survival prognostication	C-index: 0.834	IV	Multimodal AI improved prognostic stratification and survival prediction.	Limited interpretability; no prospective EV.
Kazmierski et al. [15]	2023	Multi-institutional retrospective study	2552 HNC patients + 873 EV patients	Head & Neck Oncology	ML / radiomics	OS prediction	Strong performance with EV decline	Multicenter EV	ML improved survival prediction using clinical and imaging data.	Reduced generalizability across external cohorts.
Le et al. [16]	2022	Retrospective	298 training patients + 371 validation patients	Radiation Oncology	Self-attention neural networks	Multimodal outcome prediction	AUROC > 0.70	Cross-institutional validation	Multimodal prediction of survival, recurrence, and metastasis.	Reduced generalizability across external datasets.
Qiang et al. [17]	2021	Multicenter retrospective study	3444 patients with LA-NPC	Nasopharyngeal Oncology	DL+ XGBoost	Prognostic stratification and treatment guidance	C-index: 0.719–0.757 (EV)	Multicenter EV	Improved prognostic prediction beyond TNM staging.	Retrospective despite multicenter EV.
Yu et al. [18]	2025	Retrospective	1011 patients with PTC	Thyroid Oncology	Multimodal multi-task DL	Lymph node metastasis and prognosis prediction	AUC: 0.84 (validation); 0.83 (real-world)	IV and real-world validation	Multimodal DL improved metastasis and survival prediction.	Retrospective; limited prospective validation.
Sievert et al. [21]	2024	Experimental study	12,809 CLE images from 5 patients	Oncology	GPT-4 Vision	Detection of oropharyngeal malignancy	Accuracy: 71.2%	Comparative experimental validation	Feasible CLE image classification using GPT-4 Vision.	Small cohort; inferior to experts.
Chen et al. [25]	2025	Comparative evaluation study	116 thyroid nodules from 112 patients	Thyroid Oncology	ChatGPT-4o / Claude 3-Opus	Thyroid nodule classification	AUC: 0.57	Comparative validation	Limited performance of multimodal LLMs in thyroid nodule classification.	Low accuracy; poor pathological agreement.
Mohseni et al. [26]	2025	Comparative diagnostic study	200 histopathological cases	Pathology	ChatGPT	Differential diagnosis support	Correct diagnosis in 100% of differential lists	Comparative validation	Strong performance in histopathologic differential diagnosis support.	Limited pathology spectrum; no prospective validation.
Rajendran et al. [46]	2025	AI development study	2985 HNC patients	Radiation Oncology	LLM-augmented vision transformer	Auto-delineation of radiotherapy target volumes	Superior segmentation performance	Comparative developmental validation	LLM-enhanced models improved target volume delineation.	Limited clinical implementation; workflow variability.
Dohopolski et al. [47]	2022	Retrospective	271 patients with HNC	Radiation Oncology	DL and multimodal fusion modeling	Prediction of nutritional supplementation needs during radiotherapy	AUC: 0.75	IV	Improved prediction of nutritional support needs during radiotherapy.	Single-center; retrospective; no EV.
Generative AI and Large Language Models										
Mat et al. [48]	2024	Perspective / commentary article	Not applicable	General ENT	ChatGPT / LLM	Clinical decision support	Narrative perspective	Narrative perspective	LLMs may augment, but not replace, specialist care.	Hallucinations; privacy concerns; limited domain expertise.
Edalati et al. [29]	2024	Narrative review and ethical perspective	Not applicable	General ENT	GPT-4	Clinical workflow integration and decision support	Narrative synthesis	Narrative synthesis	GPT-4 may support workflow optimization and clinical assistance.	Ethical, legal, privacy, and reliability concerns.
Shea et al. [34]	2023	Case series	Patients with delayed or complex diagnoses	General Medicine	GPT-4	Medical record analysis and diagnostic support	Exploratory evaluation	Exploratory case evaluation	GPT-4 assisted diagnostic synthesis in challenging cases.	Limited generalizability; no prospective validation.
Mete et al. [49]	2024	Comparative evaluation study	316 questions from 8 ENT in-service examinations	ENT Education	ChatGPT / Gemini / Bing	Examination performance assessment	Accuracy: 54.8%	Comparative validation	LLM performance was comparable to junior residents.	Variable reliability; limited clinical reasoning.
Javan et al. [35]	2024	Narrative review and conceptual perspective	Not applicable	General ENT	Generative AI	AI-generated clinical images and patient communication support	Conceptual perspective	Narrative conceptual analysis	Generative AI may support patient communication and education.	Inaccuracies, bias, and overreliance.

*Abbreviations:AI *Artificial intelligence, *AUROC* Area under the receiver operating characteristic curve, *AUC* Area under the curve, *BPPV* Benign paroxysmal positional vertigo, *CBCT* Cone-beam computed tomography, *CLE* Confocal laser endomicroscopy, *CNN* Convolutional neural network, *CT* Computed tomography, *DL* Deep learning, *DMFS* Distant metastasis-free survival, *DSC* Dice similarity coefficient, *ESS* Endoscopic sinus surgery, *EV* External validation, *GPT* Generative pre-trained transformer, *HD95* 95th percentile Hausdorff distance, *HNC* Head and neck cancer, *HPV* Human papillomavirus, *ICC* Intraclass correlation coefficient, *IOU *Intersection over union, *IV* Internal validation, *LA-NPC* Locoregionally advanced nasopharyngeal carcinoma, *LLM* Large language model, *LRC* Locoregional control, *ML* Machine learning, *MRI* Magnetic resonance imaging, *MSE* Mean squared error, *OPSCC* Oropharyngeal squamous cell carcinoma, *OS* Overall survival, *OSCC* Oral squamous cell carcinoma, *PET* Positron emission tomography, *PTC *Papillary thyroid carcinoma, *PTMC* Papillary thyroid microcarcinoma, *RSF* Random survival forest

Despite encouraging technological progress, translation of AI systems into routine oncologic practice remains limited. Most currently available investigations are single-center studies with heterogeneous datasets and variable methodological quality [9–18]. In addition, many predictive systems are trained using highly curated datasets that may not accurately reflect real-world clinical variability. Reproducibility across geographically diverse patient populations remains insufficiently established, while variability in imaging acquisition protocols, annotation quality, and institutional treatment pathways may further reduce generalizability [9–18]. Moreover, limited interpretability of complex neural network algorithms continues to restrict clinician trust and regulatory acceptance [31–34]. Future research should prioritize prospective multicenter validation, transparent reporting methodologies, XAI frameworks, and standardized benchmarking protocols before routine incorporation into oncologic workflows can be recommended.

## Ethical, regulatory, and clinical implementation challenges

Despite rapid technological progress, important ethical, regulatory, and implementation-related concerns continue to limit widespread incorporation of AI into otolaryngology practice [28, 29, 31–34]. Although AI systems have shown promising applications in diagnostic imaging, oncology, surgical education, and clinical decision support, important challenges persist regarding dataset quality, algorithmic transparency, physician oversight, and real-world reproducibility.

### Algorithmic bias and dataset limitations

Algorithmic bias represents one of the most important concerns in medical AI research [31–33]. AI systems are highly dependent on the quality, diversity, and representativeness of training datasets. Imbalanced datasets may lead to unequal model performance across demographic populations, potentially contributing to disparities in healthcare delivery and diagnostic accuracy. Gudis et al. emphasized the importance of avoiding bias during AI development within rhinology and otolaryngology applications [31].

In addition, dataset shift and institutional variability may substantially reduce reproducibility across healthcare systems [50]. Differences in imaging acquisition protocols, endoscopic equipment, annotation methodologies, patient demographics, and treatment pathways may limit generalizability when algorithms are applied outside the original training environment. Underdiagnosis bias has also been reported in underserved patient populations, raising concerns regarding equitable deployment of AI-supported healthcare systems [51].

Many currently available studies additionally rely on highly curated retrospective datasets collected under controlled institutional conditions. Although these datasets may optimize algorithmic performance during model development, they may inadequately reflect the complexity and heterogeneity encountered in real-world clinical practice. Consequently, broader multicenter validation remains necessary before routine clinical adoption can be recommended [50].

### Automation bias and clinical overreliance

Increasing integration of AI-supported decision systems into healthcare workflows may also introduce risks related to automation bias and excessive physician reliance on algorithmic recommendations [32, 33]. Automation bias refers to the tendency of clinicians to overtrust automated outputs while underestimating contradictory clinical findings or alternative interpretations.

Alon-Barkat and Busuioc described how selective adherence to algorithmic recommendations may influence decision-making processes in complex healthcare environments [33]. Abdelwanis et al. similarly highlighted patient safety concerns associated with automation bias in medical AI systems [32].

The emergence of large language models and generative AI platforms has introduced additional concerns regarding hallucinated outputs, fabricated references, inaccurate summarization, and misinformation [19, 29, 34]. Although systems such as GPT-4 and other multimodal large language models may provide clinically relevant support in documentation, education, and image interpretation, continuous physician supervision remains essential to ensure patient safety and prevent propagation of erroneous information.

Unlike task-specific AI systems that are developed and validated for narrowly defined diagnostic applications, large language models function as general-purpose language systems and may demonstrate variable reliability across clinical contexts. Consequently, their outputs should be interpreted as supportive rather than definitive clinical information. Current evidence supports their use in education, documentation, and information synthesis; however, independent diagnostic decision-making without physician oversight cannot presently be recommended.

AI scribes and automated documentation systems may further improve workflow efficiency and reduce administrative burden; however, inappropriate dependence on automated summaries may introduce medicolegal and patient safety concerns if inaccuracies remain undetected [19].

### Explainability and transparency

Limited interpretability of complex neural network architectures remains another major barrier to clinical acceptance of AI technologies. Many advanced AI systems function as “black-box’ models” in which the internal decision-making process remains difficult for clinicians to understand or verify.

XAI frameworks aim to improve transparency by allowing clinicians to better understand how predictive outputs and classifications are generated. Improved explainability may increase physician trust, facilitate regulatory approval, and support safer integration of AI-supported systems into routine clinical workflows [52].

Holzinger et al. proposed the System Causability Scale as a framework for evaluating the interpretability and quality of AI-generated explanations in healthcare applications [53]. Transparent algorithmic reasoning may become increasingly important as AI systems continue to expand within oncologic imaging, pathology, radiotherapy planning, and autonomous clinical decision support.

### Regulatory and medico-legal challenges

Regulatory oversight and medico-legal accountability remain incompletely defined for many AI applications in healthcare. Questions persist regarding responsibility for diagnostic errors, treatment recommendations, and adverse clinical outcomes generated or influenced by AI-supported systems.

Patient privacy and data security represent additional concerns, particularly when large datasets containing imaging, pathology, genomic, or electronic health record information are incorporated into model development. Standardization of data governance and cybersecurity practices will therefore remain essential as healthcare AI infrastructure expands.

Standardized reporting methodologies have also become increasingly important in AI research. The CONSORT-AI and SPIRIT-AI initiatives were developed to improve transparency, reproducibility, and methodological rigor in clinical investigations involving AI technologies [54]. These frameworks emphasize detailed reporting of dataset characteristics, human-AI interaction, error analysis, validation methodology, and integration into clinical workflows.

**Fig. 3:**
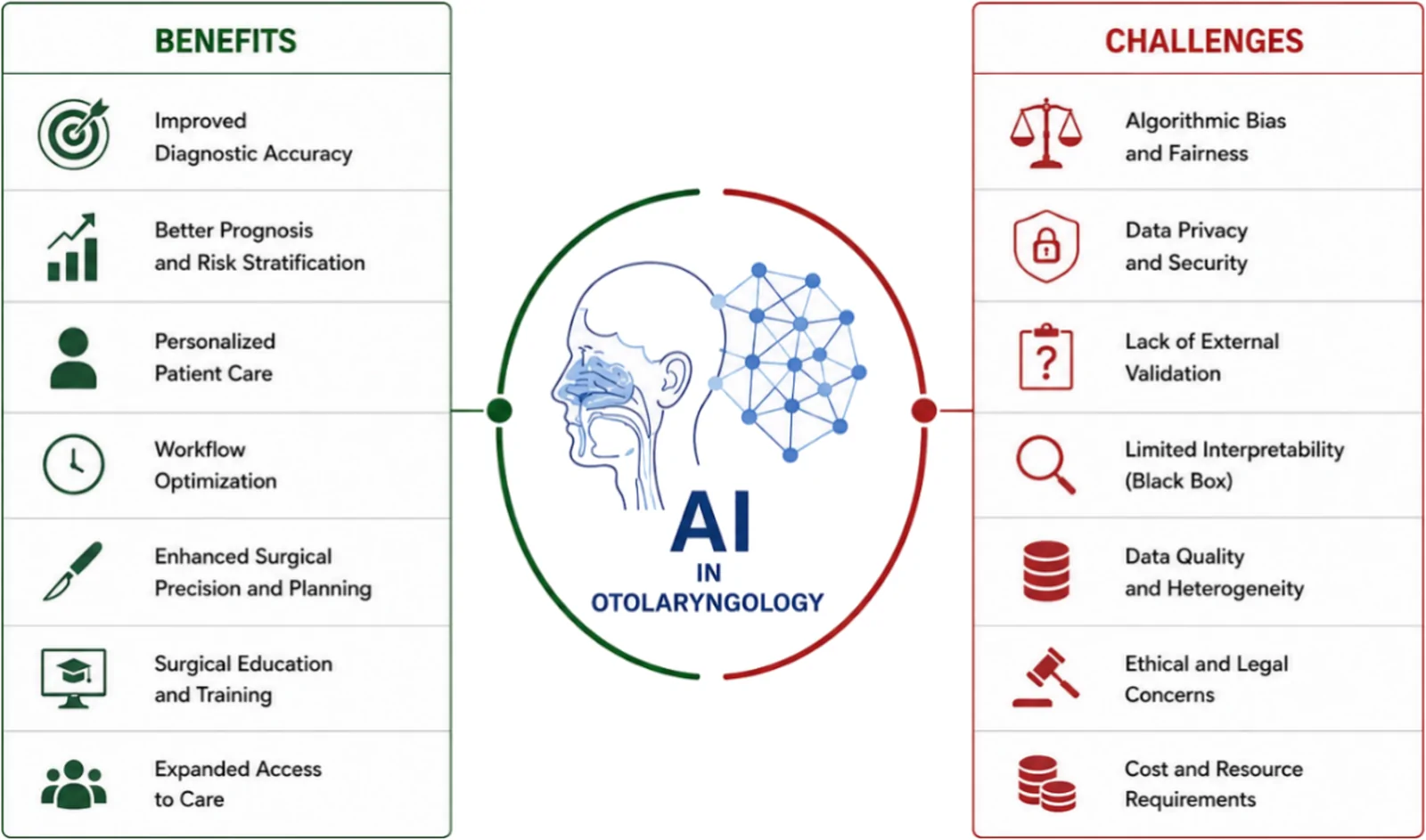
Major benefits and challenges associated with artificial intelligence implementation in otolaryngology. Potential benefits include improved diagnostic accuracy and workflow optimization, while major limitations include algorithmic bias, ethical concerns, and lack of external validation

Major benefits and current limitations associated with AI incorporation in otolaryngology are summarized in Fig. 3.

Despite ongoing challenges, AI is likely to remain an increasingly important component of future otolaryngology practice. Responsible implementation will require multidisciplinary collaboration among clinicians, engineers, data scientists, ethicists, and regulatory agencies to ensure safe, equitable, and clinically meaningful deployment of these technologies.

## Future perspectives

AI technologies are expected to play an increasingly important role in the future of otolaryngology practice [1, 2, 4]. Ongoing advances in computational power, multimodal foundation models, natural language processing, and real-time data integration may substantially expand the clinical capabilities of AI-supported healthcare systems. Future platforms will likely incorporate imaging, pathology, genomics, operative video analysis, wearable sensor data, and electronic health records into unified predictive frameworks capable of supporting precision medicine and individualized patient management [55].

In head and neck oncology, integrated predictive systems may facilitate earlier tumor detection, improved prognostic stratification, optimized radiotherapy planning, and more personalized therapeutic decision-making [9–18, 46, 47]. Similarly, future developments in rhinology, otology, vestibular medicine, and laryngology may enhance automated image interpretation, surgical navigation, hearing analysis, and diagnostic support through increasingly sophisticated computational models [3, 5–8, 20–24, 35–38].

### Emerging patterns across otolaryngologic AI research

Across otolaryngology subspecialties, several recurring patterns emerge from the current literature. First, AI systems consistently demonstrate their strongest performance in image-based tasks, including lesion detection, image segmentation, radiologic classification, and histopathologic analysis. Second, model performance frequently declines when external validation datasets are used, highlighting persistent challenges related to generalizability and institutional variability. Third, most studies remain retrospective and originate from single-center cohorts, limiting the strength of clinical conclusions. Finally, despite promising diagnostic accuracy metrics, relatively few AI systems have undergone prospective validation or achieved widespread clinical implementation. These findings suggest that the field remains in a transitional phase between technological feasibility and routine clinical adoption [1, 3, 5, 15–18, 31–34, 50].

AI-assisted diagnostic workflows may potentially contribute to earlier malignancy detection following appropriate clinical validation [56].

AI may also contribute substantially to surgical education and simulation-based training. Advanced motion analysis systems, virtual operative assistants, and AI-supported simulation environments may facilitate objective technical skill assessment, individualized trainee feedback, and standardized competency evaluation [1, 39–45]. Generative AI systems and large language models may additionally support medical education, clinical documentation, literature synthesis, and patient communication workflows [19, 29, 34].

Furthermore, several studies have reported reduced model performance when applied to heterogeneous datasets, uncommon pathologies, or external patient populations, underscoring that AI performance remains variable across clinical contexts and cannot yet be considered universally reliable [15–17, 50].

### Translational barriers to clinical implementation

Despite these advances, successful deployment of AI in real-world healthcare settings will depend on effective integration into hospital infrastructure, interoperability with electronic health records, physician acceptance, reimbursement models, and standardized regulatory oversight [31–34]. Large prospective multicenter validation studies and transparent reporting methodologies will remain essential before broader incorporation into routine clinical workflows can be recommended. The CONSORT-AI and SPIRIT-AI initiatives may provide important guidance for improving methodological rigor and reproducibility in future clinical investigations involving AI technologies.

Ultimately, AI should function as an adjunctive technology designed to augment rather than replace clinician expertise [29, 48, 55]. Human oversight, multidisciplinary collaboration, and responsible clinical governance will remain essential to ensure safe, equitable, and clinically meaningful integration of AI technologies into future otolaryngology practice.

## Conclusions

Artificial intelligence is rapidly transforming multiple aspects of otolaryngology, including diagnostic imaging, surgical education, oncologic prediction, pathology support, and clinical decision-making [1, 2, 4]. Recent developments in machine learning, generative AI, and integrated computational systems have supported increasingly validated capability across a broad range of otolaryngologic applications [9–27].

Despite ongoing technological progress, substantial challenges remain regarding reproducibility, generalizability, regulatory oversight, and integration into real-world clinical workflows [31–34].

Although many published studies report encouraging performance metrics, most remain retrospective, single-center investigations with limited external validation, and relatively few AI systems have been evaluated in prospective real-world clinical settings [1, 5, 15–18, 50].

Future progress will depend on robust real-world validation, transparent methodology, and continued physician oversight. Ultimately, artificial intelligence should be viewed as an adjunctive technology intended to augment, rather than replace, clinical expertise while improving efficiency, precision, and individualized patient care within otolaryngology practice.

## Data Availability

No new data were created or analyzed in this study.

## Abbreviations

AI: Artificial Intelligence
BPPV: Benign Paroxysmal Positional Vertigo
CI: Cochlear Implant
CNN: Convolutional Neural Network
CONSORT-AI: Consolidated Standards of Reporting Trials–Artificial Intelligence
CT: Computed Tomography
DL: Deep Learning
EHR: Electronic Health Record
ENT: Ear, Nose, and Throat
ESS: Endoscopic Sinus Surgery
GPT: Generative Pre-trained Transformer
HPV: Human Papillomavirus
LLM: Large Language Model
ML: Machine Learning
MRI: Magnetic Resonance Imaging
NPC: Nasopharyngeal Carcinoma
OSCC: Oral Squamous Cell Carcinoma
PET/CT: Positron Emission Tomography/Computed Tomography
SPIRIT-AI: Standard Protocol Items: Recommendations for Interventional Trials–Artificial Intelligence
XAI: Explainable Artificial Intelligence
